# Codeveloping a community-based, peer-led psychosocial support intervention to reduce stigma and depression among people with tuberculosis and their households in Indonesia: a mixed-methods participatory action study

**DOI:** 10.1038/s41533-024-00407-5

**Published:** 2025-01-27

**Authors:** Ahmad Fuady, Mariska Anindhita, Matsna Hanifah, Arieska Malia Novia Putri, Artasya Karnasih, Feranindhya Agiananda, Finny Fitry Yani, Marinda Asiah Nuril Haya, Trevino Aristaskus Pakasi, Tom Wingfield

**Affiliations:** 1 Department of Community Medicine, Faculty of Medicine, Universitas Indonesia, Jakarta, Indonesia; 2 Primary Health Care Research and Innovation Centre, Indonesian Medical Education and Research Institute, Faculty of Medicine Universitas Indonesia, Jakarta, Indonesia; 3 Department of Psychiatry, Faculty of Medicine, Universitas Indonesia, Dr Cipto Mangunkusumo General Hospital, Jakarta, Indonesia; 4 Department of Child Health, Faculty of Medicine, Universitas Andalas, Padang, West Sumatra Indonesia; 5 Department of Maternal and Child Health, Dr. M. Djamil General Hospital, Padang, West Sumatra Indonesia; 6 Centre for Tuberculosis Research, Departments of Clinical Sciences and International Public Health, Liverpool School of Tropical Medicine, Liverpool, UK; 7 Department of Global Public Health, Karolinska Institutet, Stockholm, Sweden; 8 Tropical and Infectious Disease Unit, Royal Liverpool and Broadgreen University Hospitals NHS Trust, Liverpool, Liverpool UK

**Keywords:** Public health, Quality of life

## Abstract

Evidence relating to peer support and community-based psychological and social (psychosocial) interventions to reduce stigma and depression among people with tuberculosis (TB) and their households is limited. This study aimed to engage with multisectoral stakeholders in Indonesia to co-develop a peer-led, community-based psychosocial intervention that is replicable, acceptable, and sustainable. We used a participatory action design and engaged key national, multisectoral stakeholders to ensure that the intervention co-design was relevant and appropriate to the TB health system and the sociocultural context of Indonesia. The co-design of the intervention evolved through four phases: (1) a scoping review to identify a long list of potential TB stigma reduction interventions; (2) a modified Delphi survey to define a shortlist of the potential interventions; (3) a national multisectoral participatory workshop to identify and pre-finalize the most viable elements of psychosocial support to distill into a single multi-faceted intervention; and (4) finalization of the intervention activities. The scoping review identified 12 potential intervention activities. These were then reduced to a shortlist of six potential intervention activities through a modified Delphi Survey completed by 22 multisectoral stakeholder representatives. At the national participatory workshop, the suitability, acceptability, and feasibility of the six potential intervention activities were discussed by the key stakeholders, and consensus reached on the final four activities to be integrated into the psychosocial support intervention. These activities consisted of: individual psychological assessment and counselling; monthly peer-led group counselling; peer-led individual support; and community-based TB Talks. In Indonesia, meaningful participation of multisectoral stakeholders facilitated co-design of a community-based, peer-led intervention to reduce TB stigma and depression amongst people with TB and their households. The intervention was considered to be locally appropriate and viable, and is being implemented and evaluated as part of the TB-CAPS intervention study.

## Introduction

Tuberculosis (TB) continues to pose a significant global health burden, causing illness in more than 10 million people and killing 1.3 million in 2023^1^. Among the myriad challenges in TB care and prevention, the pervasive influence of TB-related stigma, henceforth called TB Stigma, remains both a key psychosocial determinant and consequence of TB. TB Stigma has been demonstrated to impede people with TB-related symptoms and signs from seeking care^2,3^, to decrease adherence to TB treatment^4^, and to adversely affect the outcomes of TB treatment^5,6^. In addition to negative impacts on health outcomes, TB Stigma–often accompanied by discrimination–leads to broader negative social consequences: isolation, job and income loss, reduced quality of life (QoL), and depression^7,8^. Thus, TB Stigma has been recognised by global leaders as a formidable barrier to ending TB at both United Nations High-Level Meetings (UN HLM) on the fight against TB in 2018^9^ and 2023^10^.

Using various methods to estimate TB Stigma^6^, many studies have found that a substantial proportion of people with TB experience diverse forms of TB Stigma including enacted (experiences of being excluded, isolated, and/or discriminated against), anticipated (having perception, expectation, and/or fear of being stigmatized), or self stigma (loss of self-esteem, loss of dignity, fear and/or shame)^11–13^. In our previous study with more than 600 people with TB in Indonesia, we described that not only did 61% of people with TB experience moderate TB stigma but that such a stigma was also associated with reduced QoL and depression^7^. Importantly, people with TB in our study also identified a substantial unmet need for emotional and psychological peer support.

To design and deliver interventions to tackle TB stigma and depression, it is essential to have meaningful participation from diverse stakeholder groups. A specific stakeholder group with massive potential to combat TB the psychosocial impact of TB is peers: people with lived experience of TB who may still be on treatment or have survived TB. Peers can offer a crucial link between communities and health systems to support people with TB to seek care for their health and navigate the often-complex pathways to cure. As suggested in our previous study^7^, such peer support can be achieved by providing useful information including about TB and its diagnosis and treatment (informational support), listening to the concerns of people with TB with empathy in order to strengthen their self-esteem (emotional support), accompanying people with TB throughout their entire care journey and facilitating belonging to relevant social networks (companionship support)^14^, and support through provision of commodities and financial materials (material support)^15,16^. Leveraging the unique role of people with lived experiences of TB, henceforth called TB survivors or peers, to provide some or all of these wide forms of peer support is a promising avenue to combating the socioeconomic determinants and consequences of TB, including TB Stigma and depression.

The evidence for peer support to reduce TB Stigma and depression, especially through interventions based in the community, remains limited. However, a wealth of parallel evidence from studies that have focused on other stigmatized and stigmatizing infectious diseases also associated with mental illness including HIV/AIDS and leprosy is available^17–19^, from which the TB community can build. Moreover, to ensure that interventions to address TB Stigma are designed to be replicable, acceptable, and sustainable, necessitates the active involvement of multisectoral stakeholders, including government, civil society organization, academics, and people with TB^20^.

Therefore, extending our previous research, this study aimed to meaningfully and equitably engage relevant national stakeholders to co-develop a community-based, peer-led support intervention to reduce TB Stigma in Indonesia.

## Methods

### Study design

This study was conducted as part of the larger Medical Research Council UK funded “Community-based, peer-led intervention to reduce TB Stigma in Indonesia (TB CAPS)” implementation study. Expanding methods used in related research in the South East Asian region^21,22^, we used a participatory action research study design, which involved all relevant stakeholders in the intervention development^23,24^. This approach was used to ensure that the co-design, co-development, and subsequent delivery of the intervention was relevant and appropriate to be considered for use at scale for people with TB within the health system and sociocultural context of Indonesia^25,26^. The co-design and co-development of this community-based, peer-led intervention evolved through four phases: (1) a scoping review to identify a long list of potential TB Stigma reduction interventions; (2) a modified Delphi survey of multisectoral stakeholders to shortlist the potential TB Stigma reduction interventions; (3) a national multisectoral participatory workshop to identify and pre-finalize the most viable elements of psychosocial support to distil into a single complex intervention; and (4) finalization of the intervention activities (design and delivery).

### Scoping review

In this initial step, TB-CAPS study team members did a scoping review to identify and summarise current evidence on community-based psychosocial interventions, whether delivered by peers or not, to reduce stigma and mental health for people with TB, HIV/AIDS, and leprosy. We searched relevant articles indexed in PubMed and Web of Science, as well as WHO databases, screened their titles and abstracts, and extracted full articles and/or reports that met inclusion criteria. The full methods and findings of the scoping review are reported elsewhere^27^. The scoping review results were used to develop a long list of potential psychosocial support interventions to reduce TB Stigma among people with TB, which then informed a subsequent modified Delphi Survey and national participatory workshop.

### Modified Delphi survey

Following the completion of the scoping review, we invited relevant stakeholders with suitable and diverse TB expertise and/or experience to complete a modified Delphi survey in order to make a shortlist from the long list of activities identified in the scoping review. Delphi surveys are a group facilitation technique to transform opinion into group consensus. Many studies have also employed Delphi surveys to identify research priorities, and they are considered an essential method for developing consensual guidance and a highly informative source of evidence in healthcare research and intervention design^28–30^. Traditionally, the Delphi Survey is administered iteratively in several rounds where feedback from respondents in the previous round is shared and taken into account in subsequent rounds. Due to limited budget and timelines, we modified the Delphi survey to a single round. Despite being held in person, the survey was administered anonymously. The survey aimed to shortlist the top six potential intervention activities, not to reach a consensus—the consensus was reached through a discussion of the shortlist at the national participatory workshop explained below.

To mitigate against selection bias, we invited stakeholders representing all sectors of the TB and wider health system, the TB research community, and TB-affected communities and representatives. The relevant stakeholders included: representatives from the government (the Indonesian NTP manager, NTP managers at district levels, and healthcare workers at primary health centres responsible for TB program); TB-related civil society organizations (TB survivors organization [POP-TB] and Stop TB Partnership Indonesia); researchers (Indonesian TB researcher network [JetSet TB]; Indonesian Expert Committee for TB, Indonesian Psychiatrist Association, and Indonesian Public Health Association); and people with drug-susceptible (DS) and drug-resistant (DR) TB. The representative from each organization were required to have at least one year of related experience in the TB field. People with TB were those who had recently completed TB treatment (TB survivors) or were receiving DS- or DR-TB treatment at the time of the workshop, whose sputum had culture converted to negative and/or were considered by the National TB Programme to be non-infectious. After the TB civil society organizations sent the list of their available representatives to join this study, we reviewed the potential participant mix to ensure balance by participants’ age, gender, and suitability of expertise as far as possible.

To all invitations, we attached the explanation of the study and informed consent form. Participation in this Delphi survey was anonymous, voluntary, and unpaid. The participants could withdraw from the survey at any time.

We developed an online survey using REDCap (https://redcap.fk.ui.ac.id), which was accessible through smartphones, laptops, and other internet-enabled electronic devices. The survey included the long-list of potential psychosocial interventions that were identified in the scoping review, and all participants were asked to give a response on a Likert scale from 1 (lowest or worst score) to 4 (highest or best score) related to their perception of the intervention’s: (a) feasibility to deliver, (b) acceptability to people with TB and the health system, and (c) effectiveness to reduce TB Stigma, were it to be scaled-up to national level in Indonesia. Participants were given 20 minutes to complete the survey independently without group discussion. Members of our team were on hand to clarify any queries but did not suggest responses or influence decisions made.

After receiving all responses, we calculated the overall total scores given by all participants for each potential intervention. We then shortlisted the top six potential intervention activities based on their total scores.

### National Participatory Workshop

Following the Delphi Survey and using the shortlist of six selected intervention activities, the same participants were asked to join the national participatory workshop the next day to garner perspectives, facilitate stakeholder collaboration, and support co-creation of psychosocial interventions tailored to the Indonesian health system and sociocultural context. We engaged them in multisectoral dialogues and group work concerning the most appropriate psychosocial intervention activities to reduce TB Stigma and depression amongst people with TB in Indonesia. Based on the number of workshop participants, the participants were divided into three small groups (A, B, and C) consisting of six to seven people per group, which had been designated a priori by purposive sampling by the TB-CAPS team to ensure intra-group diversity of backgrounds and experience, and to cover all sectors including the National TB Program (NTP), District Health Officers (DHOs), public health care staff, Civil Society Organizations (CSOs), people with TB, and academics or researchers (see Supplementary Table 1). The groups were asked to discuss the potential intervention activities (either in a single activity or a combination of activities), and the technical details of the preparation, implementation, and evaluation process for the activities. Specifically, we asked groups to consider and summarise the field activities, human resources, and instruments needed to successfully implement and deliver the activities that they proposed.

At the end of the workshop, we invited all groups to present their discussion results. Research team members (AF, MA, APM) noted down the discussion points in addition to audio-recording and transcription of this session. We did not employ a qualitative analysis using the notes and records. Instead, we used the notes and audio-record as the materials that we considered in finalizing the intervention. The workshop was predominantly conducted in Bahasa language.

### Finalization of the intervention

After the participatory workshop, the proposed intervention activities that had been identified during the group work, along with the notes and recordings, were subsequently discussed between all research team members to finalize the intervention. This finalization phase was done in parallel with training module development, to which we invited a psychologist who is an expert in TB counselling and had experience in training TB peer supporters, and also representatives from national TB survivor organizations. During this process, we obtained further insights into the feasibility of the selected pre-final intervention and discussed these further to arrive at consensus of the final intervention. We then informed the Delphi Survey and National Participatory Workshop participants by email of the final selected intervention activities and design.

All participants received the Participant Information Sheet (PIS) and gave their written informed consent to join the Delphi survey and National Participatory workshop prior to the activities. This study received research ethical approval from the Research Ethical Committee of Liverpool School of Tropical Medicine (RGETEM044) and the Faculty of Medicine Universitas Indonesia (KET-1169/UN2.F1/ETIK/PPM.00.02/2023).

## Results

### Scoping review

After screening 13,252 titles and abstracts, we selected 29 articles and reports for inclusion in the scoping review. From these articles, we found 12 discrete intervention activities that were perceived by the project team to have the potential to be incorporated into a complex community-based psychosocial intervention to reduce TB Stigma and depression among people with TB in Indonesia. The detailed results of the scoping review are reported elsewhere^27^.

### Modified Delphi Survey

Of 25 persons invited to complete the modified Delphi survey, 22 participants (88%, 13 female and 9 male) completed the survey (Table 1). Three participants (a person with DS-TB, a member of the TB Civil Society Organization, and a District Health Officer) did not complete the survey, citing a lack of confidence in providing appropriate answers despite support from our team. These participants were happy to continue in other workshop activities. As planned, the participants represented diverse stakeholder groups: seven from civil society organizations including TB survivor organizations (Association of TB Patient Organizations), six from academics or researchers, five from the government (NTP, DHOs, and community health centre staff) and four people with TB (two with DS-TB and two with DR-TB).

**Table 1:** Participants in Delphi survey and national participatory workshop.

Stakeholders	Invited organisations/institutions	Number of participants	Gender
Government	The Indonesian NTP: national and district levels	2 – national 5 - district	2 F 1 M, 4 F
TB-related Civil Society Organisations*	Perhimpunan Organisasi Pasien (POP-TB) Stop TB Partnership Indonesia (STPI) Pejuang Tangguh (PETA) Jawa Barat Terus Berjuang (Terjang) Jawa Barat Konsorsium Komunita Aisyiyah (Muhammadiyah)	2 1 1 1 1	2 M 1 F 1 M 1 F 1 M
Researchers and Research Institutions/Bodies	Indonesian Expert Committee for TB (Komli-TB) Indonesian TB Research Network (JetSet-TB) Indonesian Psychiatrist Association (PDSKJI) Indonesian Public Health Association (IAKMI) Faculty of Medicine	1 1 1 1 1	1 F 1 F 1 M 1 M 1 F
People with TB	People who had completed TB treatment (TB survivors) and people who had been receiving DS-TB treatment whose sputum had converted to culture negative	2 DR-TB 2 DS-TB	1 M, 1 F 1 M, 1 F
Total		22	9 M, 13 F

*DR-TB*, drug resistant TB, *DS-TB*, drug sensitive TB, *F* female, *M* male.

*Two other Civil Society Organizations, Lembaga Kesehatan NU (LKNU) and KNCV Indonesia were invited to the workshop but were unable to attend/participate.

From the survey, we shortlisted six activities for potential inclusion in the intervention package. The activities included: group counselling, individual counselling, family counselling, home visits, training and formation of youth volunteer cadres, and religious activities. These six shortlisted activities were further discussed in the participatory workshop.

### National Participatory Workshop

The national workshop was held a day after the modified Delphi survey. Two participants from the central government could not attend the workshop. The 20 participants were divided into three groups: A, B, and C. All groups agreed that psychological counselling to cope with stigma and/or depression should be included in the intervention activities, delivered either in a group or a combination of individual and group psychological counselling. Group A suggested a combination of activities: (1) at the individual level by delivering individual psychological counselling and home visit, (2) at the family level by delivering family counselling and a home visit, and (3) at the community level by providing peer group counselling with media-assisted education, such as video, and youth volunteer activities to deliver education on TB Stigma reduction. Home visits, in particular, were proposed to be applied at treatment initiation, at the completion of the intensive phase, and at the completion of the continuation phase for all people with TB.

These multi-level activities proposed by Group A were critiqued by groups B and C as being unfeasible and highly resource-intensive. Group B reported that they had selected a more pragmatic approach by recommending only peer-led group psychological counselling, citing this as “*feasible for a relatively short period during the implementation study”* and *“not requiring high resources”*. They went on to suggest that this peer group counselling should be implemented 3-4 times over the standard 6-month DS-TB treatment period. Group B reported that this recommendation was based on evidence that mutual support groups and group counselling (as opposed to individual counselling) were more effective to reduce self-stigma and anticipated stigma, which our previous research had identified as the most potentially modifiable forms of TB Stigma.

*“Based on our previous discussions, the problems we want to address are self-stigma and anticipated stigma. Can we address TB Stigma in the community or other types of stigmas (by delivering meeting and counselling)? We need to better understand feasibility, to limit the [breadth] of problems [related to stigma] we intend to address, and to focus the intervention we develop on reducing self-stigma and anticipated stigma.” Group B representative*.

Group C offered a combination of activities consisting of (1) healthcare facility-based counselling into TB treatment including adherence and adverse effects, while people attend a routine TB visit, (2) monthly group counselling, and (3) individual counselling (when a person is indicated as having a mental health disorder). They argued that providing only group counselling means that people who do not join the meeting, some of whom may potentially have substantial unmet needs for mental health support, will not be reached.

*“(If we only provide group counselling) we cannot reach those who do not join the group counselling. Then we need help from Community Health Volunteers and TB Program Officers to identify and approach those who are not engaged (in the intervention) or do not adhere to the treatment, through medical and individual counselling.”* Group C representative

During the discussion, it was also highlighted that the intervention should not focus only on reducing TB Stigma, but also depression and anxiety.

*“Among those with a high level, or even a moderate level, of stigma, it can result in high levels of depression or anxiety (for which we have to intervene).”* Group C representative

It was recommended by all groups that, once group psychological counselling was implemented, sessions should occur at least monthly, and people with TB would ideally join 2-4 group counselling during their first six months of TB treatment. However, there were debates on who should lead and facilitate the group counselling and the optimal location for meetings to take place. Group A suggested Community Health Volunteers (CHVs) as group counselling facilitators and that meetings take place in a healthcare facility. However, Groups B and C had different opinions, suggesting that, based on some of their members’ past experiences, healthcare facilities were *“not a (physically) comfortable place”, “too cramped”, “close to a garbage dump”,* or could *“potentially lead to being recognized as having TB (by neighbours)”* that *“can lead to stigmatization”*.

Group B suggested CHVs as the lead facilitators, together with TB program officers and TB survivors. This proposal was based on experience in conducting group discussions when people with MDR-TB can chat and share experiences, called a “super group”, held once in three months, led by MDR-TB survivors. The group discussions had been implemented for more than one year in several MDR-TB facilities, funded by an international donor. They found that TB survivors had challenges to successfully facilitate group counselling, resulting in suboptimal delivery and impact. A new intervention needs to complement the available resources in the community and avoid reinventing the wheel, by only assigning TB survivor as peer group counselling leader. However, assigning TB program officers as the leading group facilitator may *“prevent people with TB from being open”* to share their thoughts.

All groups agreed that training to become a group counselling facilitator was necessary and should be implemented in the preparation phase prior to leading group sessions. Based on experiences, despite the higher proportion of female group facilitators than male, *“about 60% vs 40%”*, there was no perceived difference in acceptability and impact of counselling sessions provided by female vs male facilitators.

A training module was discussed across groups as an important prerequisite to ensure that the facilitators are well-trained prior to intervention implementation. The groups mentioned key topics that should be incorporated into the core curriculum of any training module, including TB basic knowledge, TB treatment and its adverse effects, TB Stigma, nutrition, empathy, personal communication skills, and public speaking skills. Groups noted that there were many different and complementary modes by which these topics could be taught and learnt including lectures, role-playing activities, brainstorming, and interactive games.

**Fig. 1:**
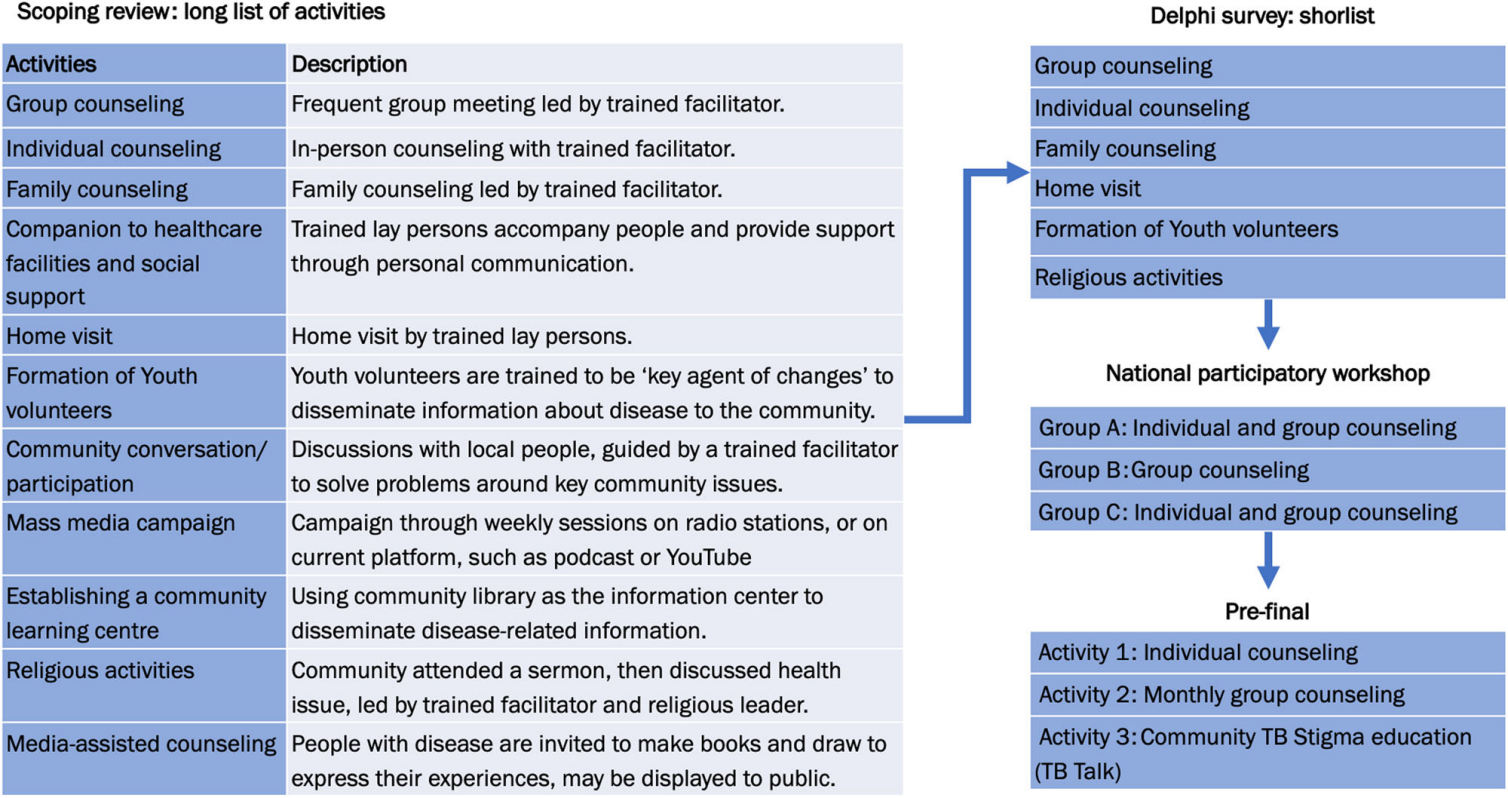
Co-development stages to select psychosocial intervention activities. Development of the intervention activities from scoping review long-list to participatory workshop Group short-list, to selected pre-final intervention activities.

At the end of the workshop, the pre-final intervention activities were formulated and updated during interactive discussions with all participants (Fig. 1). This consisted of three activities. Firstly, individual counselling by either healthcare workers or research team members applied at the first healthcare visit following a TB diagnosis. At this point, the baseline status of people with TB’s stigma levels, mental health, and quality of life will also be evaluated using tools validated in the Indonesian setting^7,12^ and a verbal and written invitation to a group psychological counselling will be provided. Secondly, a monthly community-based group counselling will be held in an agreed communal space distinct from healthcare facilities and led by TB survivors. Thirdly, a community activity, such as TB talks, will be held to improve community knowledge about TB and reduce TB Stigma in community, by inviting people with TB and their families to lead the talks and share their experiences to the public or neighbouring communities.

An important point that arose during the meeting was that people with TB participating in the workshop voiced in open discussion sessions that they felt that their experiences and perceptions were being addressed and that they were being listened to during the co-development process.

*“I didn’t ever realise that I could actually say out loud what I need as someone with TB. I have just realized that this happens now.”* Male participant with DS-TB, currently receiving treatment.

*“This is what I have been waiting for for a long time. I really wish that my friends, people undergoing drug-resistant TB treatment, could have people or someone, with whom they can share their feelings…”* A female participant and DR-TB survivor.

### Finalization of the Intervention

The pre-final intervention activities were then discussed internally amongst the research team. In this phase, we did not exclude any activities agreed upon in the workshop. Rather, based on the feasibility of these activities, we shaped the potential implementation strategy of the intervention, including the timing of activity delivery and delivery personnel including counselling providers opted to include the individual psychological counselling at the first healthcare visit to assess the acceptance to TB diagnosis, the readiness for TB treatment, and the stigma and mental health people with TB experience. This psychological counselling may also avoid *“rational non-adherence”*, when people with TB, feel that they cannot continue the treatment because of reasonable factors they face in their life, healthcare worker advice, or adverse effects—a phenomenon which was also found among people with HIV/AIDS^31^.

*“The current approach is that we want people with TB to take the treatment as soon as they are diagnosed. Once the sputum test gives a positive result, they have to start treatment directly, without assessing their readiness…. It is better to build a strong foundation (upon which people with TB accept the diagnosis and are ready for TB treatment). If they are ready, they will not discontinue the treatment.”* Psychologist

With regards to leadership of the group counselling and learning from the reported challenges of leadership by people with TB alone, especially if training is not adequate, we opted for integrated leadership and facilitation shared between TB survivors and general practitioners. We opted for TB survivors to be accompanied by general practitioners instead of TB program officers, who were reported by one stakeholder to be *“overwhelmed with their work burden at healthcare facilities”,* or CHVs, who may have a *“perception of being in a more legitimate position than people with TB”*, both potentially leading to a suboptimal counselling process. The peers, in addition, can still provide individual support through personal contact and group communication through a user-friendly communication platform, such as WhatsApp or other related platforms.

*“Based on experience, many people want to become CHVs because CHVs are perceived as occupying a legitimate social position in their community, and not necessarily because they want to accompany people with TB. I observed in X (mentioning a district name), people with TB were only ordered, even scolded, by CHVs, and it may increase stigmatization.”* Psychologist

**Fig. 2:**
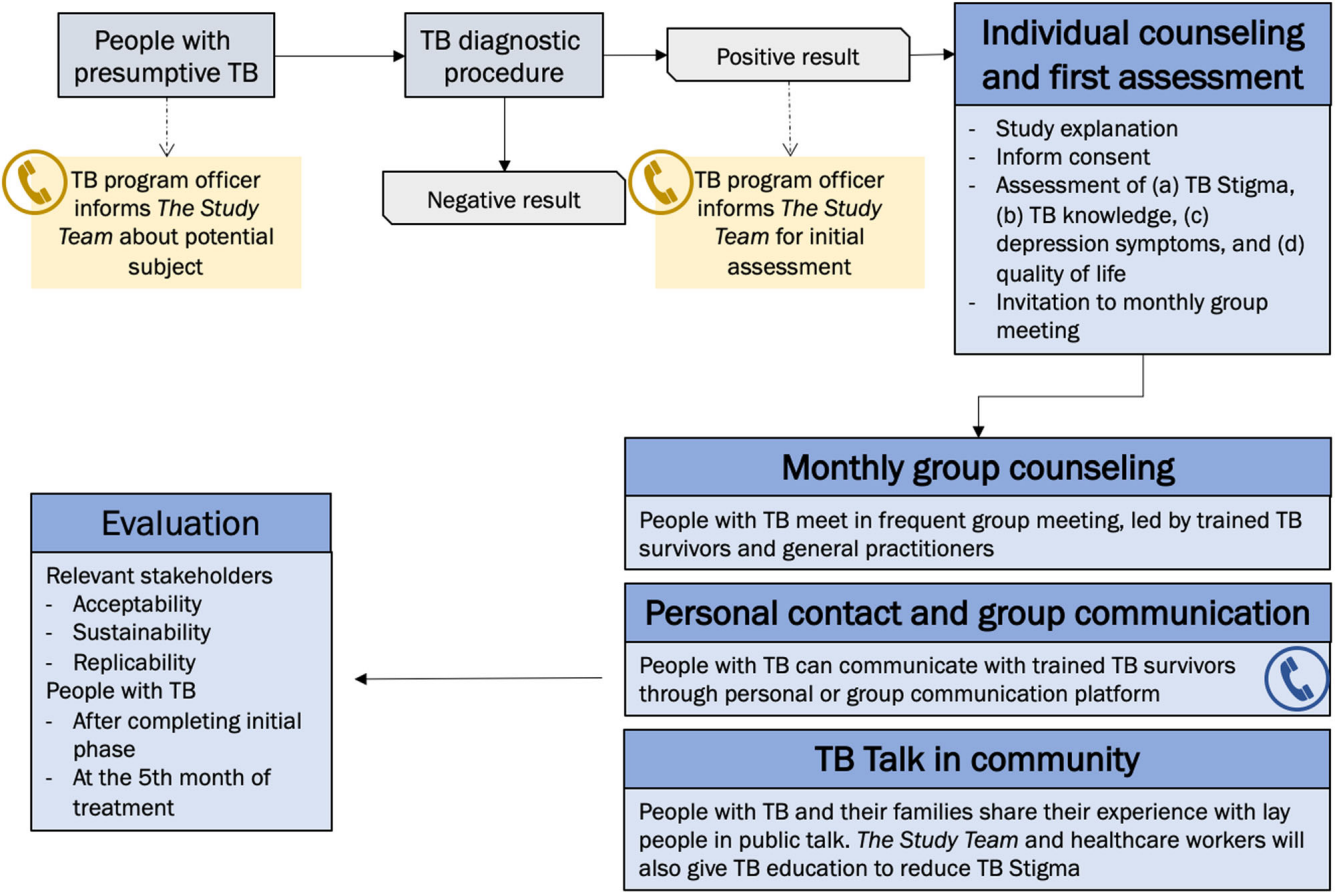
Final psychosocial intervention activities selected to be evaluated in the TB CAPS Study. Final intervention activities for the TB CAPS Study.

At finalisation, the intervention activities consisted of (1) individual counselling and initial psychosocial assessment, (2) monthly group counselling facilitated by peers/TB survivors and general physicians, (3) personal contact and group communication facilitated by peers/TB survivors, (4) TB Talks, a public education talk inviting all people in neighbouring communities, where TB survivors, people with TB, and their families share their experiences with the general community, along with public education about TB and stigma by healthcare workers and (5) evaluation. (Fig. 2)

## Discussion

This study highlights the importance of multisectoral engagement, including of TB-affected communities, to co-design psychosocial and other interventions for people with TB. During this study, a scoping review produced a long-list of interventions, a modified Delphi Process whittled those interventions into a shortlist, and a workshop-built consensus in order to co-design a single community-based, peer-led psychosocial support intervention to reduce stigma amongst people with TB through four key, inter-related activities: individual counselling and initial assessment; monthly peer-led group counselling; peer-led individual support; and community-based TB Talks. Meaningful participation at all stages but especially during the modified Delphi Survey and subsequent national workshop, afforded valuable feedback to tailor the final selected psychosocial support intervention that was perceived to be suitable, acceptable, feasible, and potentially scalable.

Community and civil society engagement in research is crucial and should encompass all relevant stakeholders, including those affected by the diseases in question, community leaders, policymakers, and healthcare workers, in order to promote equitable involvement in addressing health challenges^32^. Extensive peer-reviewed articles and grey literature reports underscore the potential benefits of community engagement, including enhancing the community’s understanding of the issues being investigated, improving researchers’ capacity to identify community needs and priorities, and emphasizing the need for culturally sensitive communication and research approaches^17,18,33,34^.

TB Stigma is a particularly sensitive issue, as evidenced by our previous study with more than 600 people with TB in seven provinces of Indonesia, which showed that 61% of people with TB experience TB Stigma, which is associated with feelings of guilt and fear and hinders those affected from disclosing their disease to others^7^. Based on these findings, we resolved to co-develop an intervention to reduce this heavy burden of TB Stigma and also mitigate associated depression due to TB in Indonesia, in a manner that was appropriate, feasible, and acceptable to people with TB, their communities, and the health system. Among various forms of community engagement in research, this study utilized two main approaches: inviting all relevant stakeholders in listing the priorities through a Delphi survey and in discussing the potential intervention through a national participatory workshop. Engaging in the early phases of the main, larger study, helped to identify issues not covered in existing literature and reports^35^. We committed to facilitate co-learning by helping all participants understand the research process and committed to have a strong partnership by inviting as diverse a group of stakeholders as possible, in terms of their backgrounds, experiences, and skills sets, in order to gain a variety of perspectives and multi-dimensional feedback^36–38^.

The involvement of people with TB in the co-development of psychosocial support to reduce stigma is aligned with the World Care Council’s “Patients’ Charter for Tuberculosis Care”^39^. The Charter specifically states that people with TB have the right to participate as “stakeholders” in the development, implementation, monitoring, and evaluation of TB research, policies, and programs. The UN HLM 2023 also enshrined the right of people with TB to “enjoy and share the benefits of scientific progress”^10^. Their involvement as peer supporters in this study is also part of their moral responsibility to share information and knowledge gained during treatment and to pass this expertise to others, including in this co-development phase. Our collaboration with the Indonesian National “Organization of Tuberculosis Survivors”, POP-TB, which is a large network of TB survivors in Indonesia, has enabled us to establish a trusted position as collaborators in developing psychosocial support interventions within their network. Through this collaboration and with healthcare workers who already engage with individuals affected by highly stigmatized TB, we developed a strategy to recruit participants experiencing high TB-related stigma, employing an approach that prioritized patience and sensitivity.

During the co-development of the intervention, people with TB voiced their personal needs, and others’ needs that they have observed during TB treatment, emphasizing the importance of the sharing and listening process for meaningful community engagement. This emphasis on listening is a crucial element in involving the community to contribute meaningfully to scientific advancements, moving beyond mere as a fulfilment of requirements stipulated by grant funders as a checkbox for community engagement throughout the study^35^. It is imperative to ensure that the intervention co-development process adheres to the fundamental principles of reciprocal and equitable relationships, demonstrating that participants are central to this collaborative development, with decision-making defined by consensus^36^. Recognizing that community engagement is an ongoing and iterative process, it is crucial to involve participants throughout all study phases, extending beyond the intervention co-development phase. Promoting this ongoing involvement as a key factor to the successful delivery of any intervention, we communicated to all workshop participants that they would be invited to subsequent phases, including during both implementation and analysis and evaluation phases^35^.

Stakeholder participants perceived that a multilevel community-based intervention that extends to individuals with TB, their households and families, and the local community can address stigma and depression, and find effective solutions simultaneously^27,40^. However, some participants raised concerns about feasibility of the intervention because of the short timeframe of the implementation project which would be a considerable challenge for implementing such multilevel intervention activities. To address this, a more feasible approach was devised but still considering activities that can foster people’s resilience against stigma and depression, encourage their treatment adherence, boost the utilization and retention of available healthcare services, and empower participants to safely disclose their disease status to supportive individuals. Initial assessments and individual counselling at healthcare facilities were deemed appropriate and feasible to identify stigma and mental health issues, and it was clearly emphasised that participation in group counselling was voluntary and without pressure. Through individual psychological counselling, TB program officers could identify and approach individuals choosing not to join group counselling but identified as having mental health problems, directing them to receive additional psychosocial support. This is a critical step since TB Stigma, as also discussed during the workshop, can result in high levels of depression and/or anxiety and the need for further relevant treatment and care^7^.

The participatory workshop, which included individuals experienced in providing psychosocial support, also aided in discerning effective and ineffective strategies. For instance, assigning TB survivors with leadership of group counselling and facilitation might result in suboptimal outcomes due to challenges with, and lack of training in, communication skills and counselling. Identifying partners to facilitate group counselling became an important step, drawing on diverse experiences and skillsets. The final intervention activities determined general practitioners as the most suitable partners to collaborate with TB survivors in facilitating group counselling. Moreover, the workshop also highlighted that whoever facilitates the group counselling should be well-trained, and the development of a training module is imperative. It is also critical to hold training of the trainers for the sustainability and replicability of the intervention in other areas. Further, there must be suitable plans in place, agreed by consensus, on appropriate remuneration for time devoted to facilitating and counselling activities.

Additionally, our findings suggested that regular communication to support people with TB and their households through accessible and user-friendly platforms, such as WhatsApp or other social media groups, is vital^27,41^. Led by TB survivors, this communication group allows individuals with TB to reach out at any time at which they encounter clinical or psychosocial problems, whether through personal lines or group channels.

This study has some limitations. First, not all invited stakeholders attended the workshop. However, the absence of three civil society organizations, which were recognized for their extensive experience in offering social support, was compensated for by representatives from alternative organizations that contributed substantial feedback. Second, the different participants’ backgrounds in each group could enrich perspectives but can also undermine the process and success of the intervention co-development because of asymmetric power dynamics amongst participants^42^. Particularly in workshops with numerous stakeholders, individuals with TB have previously reported to the study team a hesitancy to speak and experience self-stigma including feelings of inferiority, in terms of educational level, socioeconomic status, ethnicity, health, and gender. In this study, we sought assistance from POP-TB, a TB survivor organization with an extensive network in Indonesia, to identify the most suitable individuals, who can speak in public and have either personal experience of stigma or experience in support provision, to attend the workshop and share their insights. We also mitigated the potential for power asymmetry between participants by assigning one facilitator (AF, MANH, TP) in each group to guide the discussion. While facilitating the group discussion, facilitators encouraged all participants to give their opinions and reiterated the importance of mutual respect for all participants in the decision-making processes of each group. As per the modified Delphi Survey, the facilitators clarified any queries but did not give their own opinions or encourage or discourage any specific decision.

This study concluded that the participatory action mixed methods study, involving meaningful community and civil society collaboration, reached a consensus to select a community-based peer-led psychosocial intervention to reduce TB Stigma consisting of four main activities: individual counselling and initial assessment, monthly peer-led group counselling, peer-led personal support, and community-based TB Talks. The proposed intervention was considered locally appropriate, acceptable, feasible and scalable, and is now being implemented and evaluated, including for effectiveness in increasing TB knowledge, reduce TB Stigma, and improve mental health, in the Medical Research Council, United Kingdom, TB-CAPS study in two provinces of Indonesia.

## Contributions to the literature

Ensuring that holistic interventions to address tuberculosis (TB) stigma are replicable, acceptable, and sustainable, in local contexts is essential. This study actively involved multisectoral stakeholders to co-design a psychosocial intervention for TB-affected households using four critical steps: a scoping review, a modified Delphi survey, a national participatory workshop, and intervention finalization. Multisectoral engagement supports the dynamic process of co-designing and co-developing psychosocial support intervention activities to reduce TB Stigma and depression. Real-time feedback obtained during both a modified Delphi Survey and a national participatory workshop helped to refine and select the most locally appropriate psychosocial intervention tailored to the Indonesian setting. Our findings provide much-needed evidence on the use of participatory action research methods to co-design and co-develop multi-faceted interventions for TB-affected households and could be applied to other health conditions and/or in other areas of the world.

## Supplementary information

Supplementary Table 1

## Data Availability

The data of this study are not publicly available due to privacy or ethical restrictions but are available by requesting to the corresponding author.
