# Supplementary material for: Codeveloping a community-based, peer-led psychosocial support intervention to reduce stigma and depression among people with tuberculosis and their households in Indonesia: a mixed-methods participatory action study
Source: NPJ Prim Care Respir Med. 2025 Jan 27;35:7. doi: 10.1038/s41533-024-00407-5 (PMC11772848; doi:10.1038/s41533-024-00407-5)
Supplement: Supplementary file 1 — Supplementary Table 1 [file 41533_2024_407_MOESM1_ESM.docx]

**Supplementary Table 1**. Participants in Group A, B, and C at the national participatory workshop

| **Stakeholders** | **Group A** | **Group B** | **Group C** |
| --- | --- | --- | --- |
| Government including Ministry of Health | 2 (F) | 1 (F) | 1 (M), 1(F) |
| Civil society organisations | 1 (M) | 1 (M), 2 (F) | 1 (M), 1 (F) |
| Researcher, Academics | 1 (M), 1 (F) | 1 (M), 1 (F) | 1 (F) |
| People with TB | 1 (M), 1(F) | 1 (M) | 1 (M) |

**M,** Male; **F,** Female
